# Молекулярные маркеры в исследованиях генетического разнообразия представителей рода Rubus L. и перспективы их применения в селекции

**DOI:** 10.18699/VJ20.591

**Published:** 2020-02

**Authors:** А.М. Kamnev, O.Yu. Antonova, S.Е. Dunaeva, T.A. Gavrilenko, I.G. Chukhina

**Affiliations:** Federal Research Center the N.I. Vavilov All-Russian Institute of Plant Genetic Resources (VIR), St. Petersburg, Russia Altai State University, Barnaul, Russia; Federal Research Center the N.I. Vavilov All-Russian Institute of Plant Genetic Resources (VIR), St. Petersburg, Russia; Federal Research Center the N.I. Vavilov All-Russian Institute of Plant Genetic Resources (VIR), St. Petersburg, Russia; Federal Research Center the N.I. Vavilov All-Russian Institute of Plant Genetic Resources (VIR), St. Petersburg, Russia; Federal Research Center the N.I. Vavilov All-Russian Institute of Plant Genetic Resources (VIR), St. Petersburg, Russia

**Keywords:** Rubus, raspberries, blackberries, DNA markers, genetic diversity, molecular maps, MAS., Rubus, малины, ежевики, ДНК-маркирование, полиморфизм, молекулярно-генетические карты, MAS

## Abstract

Род Rubus L. (семейство Rosaceae Juss.), по оценкам разных систематиков, состоит из 12–16 подродов, объединяющих ~750 видов. Самые крупные по числу видов подроды – Idaeobatus (Focke) Focke, к которому относятся малины, и типовой подрод Rubus (=Eubatus Focke), включающий виды ежевик. Представители рода Rubus обладают высокой пищевой и хозяйственной ценностью, а также лекарственными свойствами. Селекционные исследования направлены на расширение генетического разнообразия и создание новых сортов малин и ежевик, устойчивых к биотическим и абиотическим стрессорам и отличающихся высоким качеством плодов. Современные селекционно-генетические программы все шире включают достижения молекулярной генетики и геномики. В данной статье представлен обзор фундаментальных и прикладных исследований генетического разнообразия культивируемых и дикорастущих видов рода Rubus, выполненных на основе методов молекулярного маркирования. Рассмотрены основные типы молекулярных маркеров (RFLP, RAPD, SSR, ISSR, AFLP, SCAR, SSCP, ретротранспозонные маркеры и т. д.) и области их применения в изучении представителей рода Rubus. Приведены результаты работ по использованию методов ДНК-маркирования для решения самых разных задач, включая: исследование межвидового и внутривидового генетического разнообразия, филогенетических связей видов и надвидовых таксонов, выяснение спорных вопросов систематики, генотипирование и уточнение родословных сортов малин и ежевик, изучение сомаклональной изменчивости и др. Наиболее важным результатом в практическом плане является создание насыщенных молекулярно-генетических карт для разных видов малин и ежевик, на которых локализованы многочисленные гены и QTL, детерминирующие различные хозяйственно ценные признаки. В то же время необходимо отметить, что число маркеров, перспективных для проведения эффективного молекулярного скрининга, пока еще недостаточно.

## Abstract (en)

According to estimates of various taxonomists, the genus Rubus L. (Rosaceae Juss.) consists of 12–16 subgenera comprising ~750 species. The two largest subgenera are Idaeobatus (Focke) Focke, which includes raspberries, and the type subgenus Rubus (=Eubatus Focke), which contains blackberry species. Representatives of the genus Rubus have high nutritional and economic values, as well as medicinal properties. Breeding programs are aimed at broadening genetic diversity and creating new varieties of raspberries and blackberries that are resistant to biotic and abiotic stressors and have high fruit quality. Modern breeding and genetic programs increasingly use the achievements of molecular genetics and genomics. This paper reviews the literature data on the application of molecular markers in fundamental and applied research aimed at studying the genetic diversity of cultivated and wild species of the genus Rubus. The review describes the main types of molecular markers (RFLP, RAPD, SCoT, SSR, ISSR, AFLP, SCAR, SSCP) and their application for studying the species of the genus Rubus. The results of the work on the use of DNA markers for solving different tasks are presented, including: studying the phylogenetic relationships of species, clarifying controversial issues of taxonomy, analyzing interspecific and intraspecific diversity, genotyping and pedigree analysis of raspberry and blackberry varieties, studying somaclonal variation and others. The most important applied result is the development of molecular genetic maps for raspberry and blackberry species, on which numerous genes and QTLs conferring various valuable traits have been mapped. At the same time, the number of markers that are promising for effective molecular screening is still insufficient.

## Introduction

Род Rubus L. (подсемейство Rosoideae Focke, семейство Rosaceae Juss.), по оценкам разных систематиков, состоит из 12–16 подродов, объединяющих ~750 видов (Focke, 1910, 1911, 1914; Robertson, 1974; Красовская, 2001). Са- мые крупные по числу видов подроды – Idaeobatus (Focke) Focke, к которому относятся малины, и типовой подрод Rubus (=Eubatus Focke), включающий виды ежевик. Представители рода произрастают на всех континентах, за исключением Антарктиды и аридных территорий. Наи- большее разнообразие видов подрода Rubus (=Eubatus) сосредоточено в северо-восточной Америке и Европе, а подрода Idaeobatus – в умеренной и субтропической части Восточной Азии (от Гималаев до Японии) и в западной и центральной части Китая (Бологовская, 1936). До сих пор не существует единого мнения об объеме и числе видов рода Rubus, что весьма затрудняет определение по флористическим спискам точного числа произрастающих на территории России видов. Анализ новейших региональных флористических сводок – «Конспект флоры Азиатской России» (2012) и «Флора Восточной Европы» (2001) – показал, что в Российской Федерации наибольшее видовое разнообразие подрода Rubus (53 вида) представ- лено в европейской части страны (Красовская, 2001), а подрода Idaeobatus (9 видов) – в азиатской (Овчинникова, 2012).

Три наиболее распространенных культурных вида малин – R. idaeus L. (малина обыкновенная), R. strigosus Michx. (малина щетинистая) и R. occidentalis L. (малина западная) относятся к секции Idaeanthi Focke подрода Idaeobatus (Knight, 1993). Большинство культивируемых видов ежевик входят в секции Allegheniensis, Arguti, Flagellares, Rubus, Ursini и Verotrivialis подрода Rubus (Gustafsson, 1943; Finn, 2008). Сорта малины и ежевики возделывают во многих странах. По данным на 2017 г. ведущи- ми производителями являются Мексика, Сербия, США, РФ и Польша (https://www.wikizero.com/en/Raspberry; Strik, Finn, 2012). Помимо пищевой ценности, виды малин и ежевик обладают лекарственными свойствами.

Исходно селекция малины в европейских странах основывалась на отборах и введении в культуру лесных форм R. idaeus. В ХIХ в. в Европу была завезена малина щетинистая из Северной Америки (Jennings, 1988). На- чало целенаправленной селекции малины, основанной на межвидовой гибридизации отобранных форм R. idaeus и R. strigosus, можно отнести к концу ХIХ в. Источни- ками генетического материала R. strigosus послужили всего лишь четыре сорта – ‘Cuthbert’, ‘Latham’, ‘Herbert’ и ‘Ranere’ (Daubeny, Anderson, 1989). В Европе также использовалось предельно ограниченное число сортов R. idaeus (Jennings, 1988). В большинстве межвидовых скрещиваний участвовал сорт ‘Lloyd George’ (сеянец ма- лины обыкновенной R. idaeus), который является прямым предком более 30 % селекционных сортов, выведенных до 1970 г. (Oydvin, 1970). Использование ограниченного числа одомашненных форм R. idaeus и R. strigosus на первых этапах селекционного процесса и многократное включение их в последующие скрещивания привели к резкому сокращению генетического разнообразия сортов малины, что до сих пор представляет серьезную проблему в селекции этой культуры (Jennings, 1988; Dale et al., 1993; Graham et al., 1996).

В настоящее время наиболее актуальными направлениями селекционной работы являются: создание сортов малины, устойчивых к абиотическим стрессорам, гриб- ным и вирусным болезням (например, ботритиозу, антрак- нозу, корневым гнилям, вирусу кустистой карликовости малины (RBDV – Raspberry Bushy Dwarf Virus)), и выве- дение сортов с высоким качеством плодов (Евдокименко и др., 2012). Для расширения генетического разнообразия возделываемых сортов селекционеры все большее внима- ние уделяют привлечению в гибридизацию дикорастущих видов подрода Idaeobatus, а также представителей более отдаленных видов из других подродов рода Rubus (Knight et al., 1989).

В РФ селекционные исследования, направленные на создание новых сортов малины, выполняются во Все- российском селекционно-технологическом институте садоводства и питомниководства в Москве, Кокинском опорном пункте этого института, в государственном бюджетном учреждении Самарской области «Научно-иссле- довательский институт «Жигулевские сады» в Самаре, на Свердловской селекционной станции садоводства, во Всероссийском научно-исследовательском институте садоводства им. И.В. Мичурина (Мичуринск) и на Но- восибирской зональной станции садоводства. На Алтае селекционная работа по малине ведется с 1935 г. в На- учно-исследовательском институте садоводства Сибири им. М.А. Лисавенко в Горно-Алтайске и с 1951 г. – в Бар- науле (Евдокименко и др., 2012).

В селекционно-генетических программах, направлен- ных на создание нового поколения сортов малины и еже- вики, все шире используются достижения молекулярной генетики, биотехнологии и геномики. В настоящей работе представлен обзор литературы по применению молеку- лярно-генетических методов в изучении биоразнообразия представителей рода Rubus и использованию этих под- ходов в селекционном процессе.

**Изоферментные маркеры.** Первым поколением мо- лекулярных маркеров, использовавшихся для изучения межвидового и внутривидового полиморфизма пред- ставителей рода Rubus и молекулярной паспортизации сортов, являлись различные изоферментные системы: MDH (malate dehydrogenase – малатдегидрогеназа), PGI (phosphoglucose isomerase – фосфоглюкозоизомераза), PGM (phosphoglucomutase – фосфоглюкомутаза), TPI (triosephosphate isomerase – триозофосфатизомераза), IDH (isocitrate dehydrogenase – изоцитратдегидрогеназа), SKDH (shikimate dehydrogenase – шикиматдегидрогеназа) (Cousineau et al., 1993; Kollmann et al., 2000) и EST (esterase – эстераза), PX (peroxidase – пероксидаза), LAP (leucine aminopeptidase – лейцинаминопептидаза) (Ду- наева и др., 2005). Изоферментный анализ также был применен в исследованиях типа размножения растений (половое размножение или апомиксис) (Kollmann et al., 2000). Следует отметить, что общей проблемой в исполь- зовании изоферментных маркеров является их сезонная и онтогенетическая зависимость, что ограничивает возмож-ности изучения внутривидового и популяционного гене- тического разнообразия.

Успехи молекулярной генетики привели к развитию методов ДНК-маркирования, позволяющих использовать любые ткани и органы на всех стадиях развития растений как в живом, так и в гербарном материале и анализировать не только белок-кодирующие, но и некодирующие участ- ки генома, а также повторяющиеся последовательности. В молекулярно-генетическом анализе видов рода Rubus применяют различные типы ДНК-маркеров – от ставших уже классическими AFLP, SSR, SCAR до новейших мар- керов, основанных на методах секвенирования нового поколения (NGS – next generation sequencing).

**RFLP (restriction fragment length polymorphism – полиморфизм длин рестрикционных фрагментов, ПДРФ).** В целях изучения межвидового и внутривидового генети- ческого разнообразия представителей рода Rubus RFLP- маркеры начали применять еще в 90-х гг. прошлого века, когда информация о нуклеотидных последовательностях геномов видов малин и ежевик отсутствовала. Поэтому в качестве зондов использовали пробы чужеродной ДНК, например фрагменты ДНК фага М13 (Nybom et al., 1990; Nybom, Hall, 1991; Hoepfner et al., 1993), пробы мини-са- теллитной ДНК гена миоглобина человека (Parent, Pagé, 1992), а также пробы из библиотек хлоропластной ДНК томатов (Moore, 1993) и ячменя (Waugh et al., 1990). Про- бы чужеродной ДНК были успешно использованы для генотипирования сортов малины и ежевики и анализа их родословных (Nybom, Hall, 1991; Parent, Pagé, 1992; Moore, 1993); исследования генетического разнообразия популяций R. idaeus, растущих на загрязненных и эколо- гически чистых территориях (Keane et al., 1998); изучения сомаклональной изменчивости сортов малины в культуре in vitro (Hoepfner et al., 1993) и в анализе полиморфизма потомства разных видов ежевик, способных к апомиктич- ному размножению (Kraft, Nybom, 1995; Kraft et al., 1996; Nybom, 1998; Werlemark, Nybom, 2003).

**RAPD (random amplification of polymorphic DNA – случайно амплифицированная полиморфная ДНК).** Поскольку работа с RAPD-маркерами не требует информации о нуклеотидных последовательностях, практически у всех культур, в том числе у малин и ежевик, это были первые из применяемых ПЦР-маркеров. Наиболее ак- тивно их привлекали для генотипирования и уточнения родословных селекционных сортов: малины обыкновен- ной (Graham et al., 1994; Stafne et al., 2003; Umar et al., 2010; Simlat et al., 2018), малины западной (Weber, 2003) и ежевики (Stafne et al., 2003; Каган и др., 2014), а также дикорастущих популяций R. idaeus (Graham et al., 1997).

В работе корейских исследователей RAPD-анализ был применен для уточнения происхождения местного сорта KСВ (Korean Cultivated Bramble – ежевика, культивируемая в Корее) (Eu et al., 2008). Результаты указывают на внутрисортовую гетерогенность KСВ, большинство образцов которого (13 из 14) кластеризовались вместе с образцами видов R. occidentalis и R. crataegifolius Bunge, и только один образец KСВ попал в один кластер вместе с R. coreanus, который ранее считался его предком.

В целом многие авторы отмечают, что метод RAPD- анализа отличается нестабильностью и слабой воспроиз- водимостью результатов, поэтому на современном этапе предпочтение отдается другим типам маркеров.

**AFLP (amplified fragment length polymorphism – полиморфизм длины амплифицированных фрагментов).** Высокополиморфные AFLP-маркеры часто привлекались в исследования генетического разнообразия представите- лей рода Rubus. В работе Miyashita et al. (2015) их исполь- зовали для филогенетического анализа образцов разных видов рода Rubus, растущих на различных японских островах. Материал включал сорта малины обыкновенной и ежевики, образцы дикорастущих популяций R. idaeus var. aculeatissimus и образцы 10 диких видов, относящихся к четырем подродам: Anoplobatus, Eubatus, Malachobatus и Idaeobatus. Образцы выборки разделились на семь клад. Виды, относящиеся к первым трем подродам, сформи- ровали три отдельные клады, тогда как виды подрода Idaeobatus разделились на четыре кластера. Интересно, что образцы R. idaeus var. aculeatissimus, собранные на о. Хоккайдо, оказались более гетерогенны (уровень поли- морфизма 95.0 %), чем образцы популяций дикорастущих видов R. crataegifolius, R. parvifolius и R. phoenicolasius Maxim., которые были собраны на трех островах – Хон- сю, Кюсю и Хоккайдо. На основании результатов AFLP- анализа авторам удалось отделить дикорастущие образцы R. idaeus от сортов малины обыкновенной (Miyashita et al., 2015).

J. Kollmann с коллегами (Kollmann et al., 2000), изучив внутривидовую генетическую изменчивость европейских видов R. armeniacus Focke и R. bifrons Vest., сделали вывод о том, что уровень полиморфизма зависит прежде всего от типа размножения растений (апомиксис или половое размножение). Этот вывод подтвердил и M.L. Marulanda (Marulanda et al., 2007), изучавший генетическое раз- нообразие культурных и диких видов малин и ежевик из региона Центральных Анд Южной Америки (R. glaucus Benth., R. adenotrichos Schltdl., R. bogotensis Kunth, R. robustus C. Presl., R. rosifolius Sm., R. urticifolius Poir.).

С помощью AFLP-маркеров была исследована генети- ческая структура популяций инвазивного вида R. alceifolius Poir. и восстановлена история его интродукции из Юго-Восточной Азии, где выявлен максимальный уровень меж- и внутрипопуляционной изменчивости, в Австралию (низкий уровень полиморфизма) и на острова Индийского океана, на которых популяции часто представлены еди- ничными AFLP-генотипами (Amsellem et al., 2000).

Турецкие исследователи применили AFLP-маркеры для изучения генетического разнообразия дикорастущих образцов малины R. idaeus, собранных в разных регионах Турции (Ercisli et al., 2008), и селекционного генофонда ежевик (Ipek et al., 2009; Agar et al., 2011). В этих работах продемонстрирован высокий уровень полиморфизма местных популяций. Авторы отметили, что полиморфные селекционные клоны, отобранные по признакам качества и устойчивости к болезням из дикорастущих популяций ежевики кавказской – R. сaucasicus L. (Agar et al., 2011) и малино-ежевичных гибридов (R. idaeus × R. ursinus – бойзенова ягода) (Ipek et al., 2009), довольно сильно отличались от широко распространенных в Турции генетически однородных североамериканских селекционных сортов ежевики.

**Маркеры, основанные на микросателлитных повторах.** Для выявления полиморфизма микросателлитных локусов наиболее часто используют SSR- и ISSR-маркеры (SSR, simple sequence repeats – простые повторяющиеся последовательности; ISSR, inter simple sequence repeats – межмикросателлитные последовательности). В первой работе по изучению SSR-полиморфизма у представи- телей рода Rubus микросателлитные участки выявляли методом блот-гибридизации по Саузерну, используя в качестве зонда две синтетические ДНК-пробы с тандемно повторяющимися последовательностями GACA и GATA (Bussemeyer et al., 1997).

В настоящее время праймеры для SSR-анализа раз- рабатывают на основе информации о фланкирующих микросателлитные повторы участках. Для этого проводят поиск повторов в известных последовательностях или в сиквенсах, полученных в экспериментальных исследо- ваниях. Например, в работе J.M. Bushakra с соавторами (Bushakra et al., 2015b) по результатам секвенирования библиотек кДНК были разработаны наборы SSR-маркеров для R. idaeus (сорт ‘Heritage’) и R. occidentalis (сорт ‘Bristol’) – 131 и 288 пар праймеров соответственно.

Существует, однако, и другой способ поиска микро- сателлитов, не требующий информации об анализируе- мом геноме, с использованием библиотек, обогащенных SSR-повторами. Геномную ДНК подвергают воздействию рестриктаз, после чего рестрицированные фрагменты клонируют в BAC-векторах (BAC, bacterial artificial chromosome – искусственная бактериальная хромосома). Полученные библиотеки BAC-клонов скринируют путем гибридизации с олигонуклеотидными пробами, например (GA)20, (CA)20, (AT)20, (GC)20, (AGC)15 и др. Позитивные ВАС-клоны, содержащие соответствующие микросател- литные участки, отбирают, секвенируют и, основываясь на информации об их последовательностях, разрабаты- вают SSR-праймеры. Подобный подход для малины был применен в ряде работ (Graham et al., 2002, 2004, 2006; Lopes et al., 2006; Lee et al., 2015). В частности, J. Graham с соавторами (Graham et al., 2002) выявили SSR-районы путем секвенирования клонов из библиотек, обогащенных последовательностями (AC)n и (AG)n. Разработанные праймеры были апробированы на выборке из 50 гено- типов, включающей сорта малины обыкновенной и ма- лины западной, сорта ежевики, образцы дикорастущих видов R. grabowski, R. deliciosus и межвидовые гибриды. В итоге было отобрано десять наиболее информативных пар праймеров, генерирующих у изученных образцов значительное число (от 7 до 16) полиморфных продуктов (Graham et al., 2002).

В дальнейшем многочисленные группы исследовате- лей создавали наборы SSR-маркеров для разных видов малин и ежевик: R. hochstetterorum (Lopes et al., 2006), R. occidentalis (Dossett et al., 2012a), R. coreanus (Lee et al., 2015), R. glaucus (López et al., 2019), а также сортов ежевики (Lewers et al., 2008). В ряде работ были разра- ботаны мультиплексные системы (Fernández-Fernández et al., 2011; Zurn et al., 2018), разрешающую способность которых проверяли на разных моделях. Так, J. Zurn et al. (2018) апробировали мультиплексную систему из шести пар SSR-праймеров в эксперименте по детекции генети- ческого материала родительских сортов у 489 F1 сеянцев ежевики из 18 комбинаций скрещиваний. По результатам SSR-анализа 94.5 % потомков были признаны гибрида- ми F1, остальные генерировали фрагменты, отсутствую- щие у родительских сортов, и были отнесены к категории “off-cross” – с апомиктичным способом размножения. С использованием мультиплексной системы из восьми пар SSR-праймеров были генотипированы полученные из разных генбанков 177 образцов ежевик, среди которых выявлены дублеты (Zurn et al., 2018).

M. Woodhead с соавторами (Woodhead et al., 2008) про- вели поиск EST-SSR-маркеров (EST, expressed sequenced tag – экспрессирующиеся последовательности) в библиотеках кДНК, полученных из корней и почек сортов ‘Glen Moy’ (R. idaeus), ‘Latham’ (R. strigosus) и потомков от их скрещивания в количестве 188 сеянцев. Полиморфизм EST-последовательностей был ассоциирован с измен- чивостью следующих признаков: срок распускания по- чек, мощность растений, устойчивость к болезням. По результатам данного исследования идентифицированы участки псевдохромосом, связанные с признаками каче- ства плодов и устойчивостью к фитофторозной корневой гнили малины. Значительная часть EST-SSR-праймеров, разработанных для селекционных сортов малины обыкно- венной (R. idaeus), была способна к амплификации ДНК у ряда диких видов рода Rubus, что, по мнению авторов, указывает на наличие у них функциональных аллелей генов, контролирующих хозяйственно ценные признаки, и на перспективы вовлечения этих видов в селекционный процесс (Woodhead et al., 2008).

Созданные наборы SSR-маркеров широко использо- вались для изучения генетического разнообразия и ге- нотипирования селекционных сортов ежевики и малины (Castillo et al., 2010a; Лебедев и др., 2018), малины обык- новенной (Badjakov et al., 2009; Lamoureux et al., 2011; Girichev et al., 2015; Lacis et al., 2017) и малины западной (Dossett et al., 2012а, b).

N. Castillo с коллегами (Castillo et al., 2010a) проанализи- ровали 48 сортов малины и 48 сортов ежевики с помощью 13 пар SSR-праймеров, одна из которых была разработана на основе последовательности из GenBank Национального центра биотехнологической информации США (National Center for Biotechnological Information, NCBI (http://ncbi.nlm.nih.gov), а остальные – на базе геномных библиотек сорта малины ‘Meeker’ и сорта ежевики ‘Marilon’. По результатам кластерного анализа полиплоидные (4х – 10х) сорта ежевики разделились на два основных кластера согласно их принадлежности к различным селекционным программам, реализуемым на востоке и западе США. Авторы объясняют полученные результаты использова- нием селекционерами восточных и западных штатов в интрогрессивной гибридизации различных диких видов разного уровня плоидности (Castillo et al., 2010a). Обособленные группы были сформированы сортами, сходными по происхождению (созданными с участием R. strigosus, R. idaeus или межвидовых гибридов), а также сортами, имеющими общий признак – способность к плодоноше- нию на побегах первого года (Castillo et al., 2010a).

С помощью SSR-маркеров проведено генотипирова- ние российских сортов малины обыкновенной и сортов селекции сопредельных стран (Lamoureux et al., 2011; Lacis et al., 2017), а также европейских сортов (Girichev et al., 2015). В этих исследованиях не было получено четкой кластеризации сортов, созданных в селекционных программах разных стран, и сортов, имеющих различное генетическое происхождение.

Другая область применения SSR-маркеров связана с изучением генетического разнообразия дикорастущих популяций разных видов малин и ежевик: R. idaeus (Graham et al., 2009), R. mollucanus L. (Bussemeyer et al., 1997), R. crataegifolius, R. fruticosus L., R. coreanus Miq. (Lee et al., 2016), аборигенных ежевик Кении (Ochieng et al., 2018). Анализ разнообразия дикорастущих в Шотландии популяций R. idaeus (Graham et al., 2009) выявил высокий уровень генетического разнообразия: 10 пар SSR-праймеров генерировали 80 аллелей у изученных образцов 12 популяций. Примечательно, что только 18 из них были выявлены у культивируемых образцов малины обыкновенной, что указывает на необходимость расширения генетического разнообразия сортов, в том числе за счет привлечения в скрещивания образцов изученных дикорастущих популяций, и, следовательно, указывает на важность их in situ сохранения.

Обратная ситуация выявлена при исследовании по- лиморфизма дикорастущих популяций R. occidentalis, собранных в 27 штатах США и в двух канадских провин- циях. Оказалось, что у дикорастущих популяций генети- ческое разнообразие было ниже, чем у культурных форм малины западной, поэтому, по мнению авторов, данные природные популяции для дальнейших селекционных работ не представляют большого интереса (Dossett et al., 2012b).

SSR-маркеры использовали также для оценки генети- ческой стабильности криорегенерантов малин и ежевик (Castillo et al., 2010b). Авторы сравнивали молекулярные спектры исходных растений, криорегенерантов и ex vitro растений с помощью 10 пар SSR-маркеров и 10 пар AFLP- праймеров. SSR-маркеры не обнаружили различий между анализируемыми генотипами, тогда как AFLP-маркеры позволили выявить полиморфизм у криорегенерантов. Однако после одногодичного выращивания в полевых условиях криорегенеранты оказались идентичны ис- ходным растениям. Выявленный у криорегенерантов полиморфизм авторы связывают с эпигенетической из- менчивостью.

Настоящий прорыв в разработке микросателлитных маркеров связан с появлением методов секвенирования нового поколения (NGS): SSR-локусы стало возможно выявлять в полногеномных сиквенсах. Так, на основании более 40 миллионов коротких прочтений последователь- ностей видов R. occidentalis и R. idaeus было выявлено около 6000 SSR-локусов (Dossett et al., 2015), из которых для анализа отобрано 288 (по 144 для каждого вида). По- казано, что праймеры, разработанные на базе сиквенсов малины западной, оказались более специфичными для этого вида: их амплификация у образцов R. occidentalis проходила более эффективно по сравнению с праймерами, разработанными на базе сиквенсов R. idaeus. В то же время для малины обыкновенной источник последовательностей при создании праймеров значения не имел. Всего было отобрано 166 пар SSR-праймеров, детектирующих как внутривидовой, так и межвидовой полиморфизм (Dossett et al., 2015).

Для разработки ISSR-маркеров не требуется информации о геномных последовательностях у изучаемых объектов. Анализ образцов дикорастущей малины (R. idaeus) из 19 пунктов Черноморского побережья Турции, проведенный при помощи 15 ISSR-праймеров, показал перспективность всех апробированных в этой работе маркеров. Наилучшие результаты (высокий полиморфизм, PIC > 0.3) показали праймеры (GGGT)4, BDB(CA)8 и (AG)8YC (Cekic et al., 2018). Работа В.В. Соболева и коллег (2009) была направлена на генотипирование российских сортов малин (15 ремонтантных и 12 неремонтантных) и образцов пяти видов малин. Исследуемые образцы раз- делились по группам на ремонтантные сорта и сорта с лет- ним типом плодоношения. По результатам опыта высказа- но предположение, что один из ISSR-маркеров (а именно К19 с последовательностью (AC)8YA) ассоциирован с генетическим(и) фактором(-ами), детерминирующим(и) признак ремонтантности (Соболев и др., 2009).

**SSCP (single-strand conformation polymorphism – полиморфизм конформации одноцепочечной ДНК).** SSCP-анализ основан на изменении конформации одно- нитевой ДНК при мононуклеотидных заменах, т. е. при наличии однонуклеотидного полиморфизма. Изменение конформации ДНК, в свою очередь, приводит к измене- нию подвижности ПЦР-продукта в полиакриламидном геле относительно амплификатов у исходного генотипа. Известна работа корейских исследователей, в которой вы- яснялось происхождение культивируемого в Корее мест- ного сорта KСВ (=Korean Cultivated Bramble) от одного из видов – R. coreanus или R. occidentalis. По результатам SSCP-анализа трех межгенных спейсеров пластидной ДНК (atpB~rbcL, trnT~trnL и trnL~trnF) образцы KСВ с большей вероятностью относятся к виду R. occidentalis, чем к R. coreanus (Eu et al., 2010).

**SCAR (sequence characterized amplified region – амплифицированная область с известной нуклеотидной последовательностью).** В зависимости от уровня специфичности SCAR-маркеры чаще всего используют для идентификации определенных аллелей генов (например, доминантных аллелей R генов устойчивости, см. далее раздел MAS (marker-assisted selection – маркер-вспомога- тельная селекция), а также для детекции индивидуальных хромосом, построения генетических карт, выявления ге- нетического материала определенных видов. Например, SCAR-маркеры, разработанные на основе специфичных RAPD-фрагментов R. caesius L., позволили выявить в коммерческих образцах ладанника Cistus incanus L. от 0 до 2 % примесей растительного материала данного вида ежевики (Marieschi et al., 2010). В рамках селекционной программы канадской провинции Квебек с использова- нием SCAR-маркеров проведена идентификация сортов малины обыкновенной и малины пурпурной (гибрид R. idaeus и R. occidentalis) (Parent, Pagé, 1998).

**Ретротранспозонные маркеры.** В отличие от дру- гих культур, для представителей рода Rubus работ по ретротранспозонным маркерам известно немного. Так, Y. Liang с коллегами (Liang et al., 2016), изучая LTR-последовательности (LTR, long terminal repeat – длинный концевой повтор) в геноме черемухи, разработали 336 пар праймеров, часть из которых была способна амплифи- цировать ДНК сортов малины и ежевики – 8.6 и 6.5 % соответственно. Отметим, что в GenBank депонированы некоторые последовательности транспозонов малины, например ретротранспозон Cassandra (https://www.ncbi.nlm.nih.gov/nuccore/AY860317).

**ДНК-штрихкодирование.** Для ДНК-штрихкодирования у растений чаще всего используют последовательности внутренних транскрибируемых спейсеров рибосомальной ДНК (ITS1 и ITS2; ITS – internal transcribed spacer) и раз- личные локусы пластидной ДНК (Шнеер, Родионов, 2018; Saddhe, Kumar, 2018). Методы ДНК-штрихкодирования только начинают применять в изучении сложного рода Rubus, формирование генетического разнообразия кото- рого обусловлено процессами межвидовой гибридизации, различными формами размножения растений (половое, вегетативно-клональное, агамное, включая апомик- сис) и полиплоидии (хромосомные числа варьируют от 2n = 2x = 14 до 2n = 12x = 84) (Jennings, 1988; Красовская, 2001; Meng, Finn, 2002). Вопросы таксономии и филоге- нетических отношений видов рода Rubus требуют отдель- ного обсуждения; в настоящей работе мы остановимся лишь на некоторых исследованиях, в которых методы генетического баркодинга использовали для определения границ и изучения таксономического статуса подродов, секций, видов.

L.A. Alice и C.S. Campbell (1999) провели секвени- рование и сравнительный анализ последовательностей ITS1 и ITS2 для 56 видов из 12 подродов рода Rubus, а также 5 видов рода Dalibarda L. (в других системах Dalibarda рассматривают как подрод рода Rubus). Ре- зультаты молекулярно-филогенетических исследований не согласуются с традиционным разделением рода Rubus на 12 подродов. На филогенетическом древе изученные виды четко разделились на четыре группы. Первая клада содержала все изученные виды 9 подродов; в отдельную группу с высокой поддержкой выделились виды еще трех подродов Comaropsis, Dalibarda и Lampobatus, произрастающие в Южном полушарии; третья клада включала виды подрода Rubus и R. alpinus из подрода Lampobatus. Монофилетичную кладу сформировали только виды под- рода Orobatus (Alice, Campbell, 1999).

В работе Y. Wang et al. (2016) изучен полиморфизм ядер- ных ITS- и пластидных (rbcL, rpl20-rps12, trnG-trnS) по- следовательностей для проверки гипотез о происхождении видов малин и ежевик, произрастающих в Китае. Показа- но, что подрод Malachobatus является монофилетическим, а подроды Idaeobatus и Cylactis – полифилетическими. Выявленные в данной работе несоответствия в топологии дендрограмм, построенных по данным о полиморфизме ядерных генов рРНК и участков пластидного генома, указывают на гибридогенное происхождение изученных видов из секций Rosaefolii, Leucanthi, Corchorifolii (Wang et al., 2016).

A.J. Fazekas с коллегами (Fazekas et al., 2008) проана- лизировали последовательности 9 локусов органельных ДНК у малины обыкновенной, малины западной, ежевики аллеганской (R. allegheniensis L.) и малины душистой (R. odoratus L.). Были апробированы 8 локусов пластидной ДНК, а также митохондриального гена cox1. Большинство (6 из 8) изученных локусов не поддерживали гипо- тезу о монофилетическом происхождении видов малин, исключение составили два локуса – ген matK и спейсер atpF/ atpH (Fazekas et al., 2008).

Результаты цитированных выше работ указывают на необходимость таксономической ревизии рода Rubus.

**WGS и SNP (whole genome sequencing – полногеномное секвенирование, single nucleotide polymorphism – однонуклеотидный полиморфизм). ** В последнее время методы высокотехнологичного секвенирования стали при- влекаться и в исследования видов рода Rubus. Насколько нам известно, на сегодня существуют полногеномные сиквенсы двух видов этого рода – R. idaeus (Wight et al., 2019) и близкого ему R. occidentalis (VanBuren, 2016). С помощью современных технологий секвенирования и генетического анализа выявлен высокий уровень синте- нии геномов R. occidentalis и земляники лесной Fragaria vesca L. (VanBuren, 2016).

Полногеномное секвенирование позволяет одновременно генерировать огромное количество SNP-маркеров, которые применяют для создания генетических карт (Ward et al., 2013; Hackett et al., 2018), идентификации генов устой- чивости к патогенам, например к Verticillium (VanBuren et al., 2018), а также для картирования хозяйственно ценных признаков (López et al., 2019). В работе J. Ryu et al. (2018) SNP-маркеры были использованы и для регистрации му- тационных изменений, индуцированных воздействием гамма-лучей, у растений 14 образцов ежевик и гибридов Boysenberry.

**Microarray технологии (ДНК-чипы). **Известно не- сколько работ, выполненных на малине с применением данной технологии. В одной из них изучали экспрессию генов с целью выяснения причин остановки процессов жизнедеятельности в почках у сорта малины ‘Glen Ample’ (Mazzitelli et al., 2006). Было выявлено свыше 80 генов, имеющих отношение к клеточному метаболизму, транскрипционным процессам и др. и, вероятно, регули- рующих процесс распускания почек. T.P. Gotame et al. (2014) изучали экспрессию генов у четырех сортов малины обыкновенной в зависимости от температуры окру- жающей среды. Авторы идентифицировали 644 гена, экспрессия которых зависела от температурных условий, но из них только 12 транскрибировались у всех четырех сортов. Работа G.E. Fernandez (Fernandez et al., 2018) была направлена на выяснение генетических механиз- мов возникновения белых костянок в плодах ежевики. Выявлено более 12 тыс. генов, дифференциально экс- прессирующихся в черных и белых костянках. Оказалось, что количество РНК в белых костянках существенно снижено по сравнению с черными; в качестве причины возникновения данного феномена авторы рассматривают супрессию генов, ответственных за биосинтез нуклеино- вых кислот, в ответ на воздействие стрессовых условий (Fernandez et al., 2018).

**Использование ДНК-маркеров для построения генетических карт.** Первая карта генома R. idaeus была сконструирована на основании анализа расщепляющейся популяции потомков от скрещивания двух сортов: ‘Glen Moy’ (R. idaeus) и ‘Latham’ (R. strigosus). Изначально на карту было нанесено 30 SSR-, 4 EST- и 206 AFLP-маркеров. На полученных группах сцепления были картированы локусы количественных признаков (QTL, quantitative trait loci – локусы, вовлеченные в контроль количествен- ных признаков), таких как «наличие шипов на побегах», «плотность и распределение корневых волосков» (Graham et al., 2004). В дальнейшем эта карта совершенствовалась с привлечением новых ДНК-маркеров (Graham et al., 2006, 2015; Raluca et al., 2006; Woodhead et al., 2008; Simpson et al., 2017). По мере насыщения карты маркерами на ней были локализованы многие гены и QTL хозяйственно ценных признаков, например ген опушения побегов Н, ответственный также за устойчивость малины к ряду заболеваний (в том числе к серой гнили побегов), гены и QTL устойчивости к пятнистости побегов (группы сцепления (linkage groups) LG2 и LG4), ржавчине (LG3 и LG5) (Graham et al., 2006), а также к вирусам пятнистости листьев и хлороза сосудов (LG2 и LG7) (Raluca et al., 2006). Разработанная карта была также использована для поиска в геноме малины QTL, отвечающих за синтез антоцианов; соответствующие участки были найдены в группах сцепления LG1 и LG4 (Kassim et al., 2009). В работе J. Graham et al. (2015) были картированы QTL, связанные с признаком крошащихся плодов; данные ло- кусы были обнаружены в группах сцепления LG1 и LG3. Из последних исследований упомянем (Simpson et al., 2017), в котором преимущественно в группе сцепления LG3, а также LG1 и LG5 выявлены QTL, ответственные за созревание и размягчение плодов.

M. Woodhead et al. (2010) анализировали ту же самую популяцию ‘Glen Moy’ × ‘Latham’ с использованием 43 пар праймеров, часть из которых была разработана на основе последовательностей генов рода Prunus L. В результате были картированы 15 QTL, ассоциированных с призна- ками созревания плодов и устойчивостью к ряду пато- генов. Позднее в этой же популяции были картированы QTL, ассоциированные с признаком ветвления побегов, и изучены вклады генетической и экологической состав- ляющих в формирование данного признака (Woodhead et al., 2013). Фенотипизацию проводили в различных усло- виях окружающей среды: в теплице, в поле и в условиях заражения растений Phytophthora rubi – возбудителем корневой гнили малины. Длившийся на протяжении шести лет эксперимент позволил картировать несколько QTL, вовлеченных в контроль ветвления побегов, в группах сцепления LG2, LG3, LG5 и LG6, причем в LG3 и LG5 выявлена колокализация с QTL, влияющим на мощ- ность растений, а в LG6 – с QTL устойчивости к корневой гнили.

Другая карта была создана в работе D.J. Sargent et al. (2007) на основе результатов косегрегационного анализа 95 AFLP- и 22 SSR-маркеров в потомстве, полученном от скрещивания сортов малины ‘Malling Jewel’ и ‘Malling Orion’. На этой карте были локализованы ген А1, контро- лирующий устойчивость к малинной тле (LG3), и ген dw, ответственный за карликовый габитус растения (LG6).

В исследования по молекулярному картированию во- влекались и другие виды рода Rubus. Так, J.M. Bushakra et al. (2012) сконструировали карту сцепления маркеров для гибридной популяции, полученной от скрещивания образцов малины западной и малины обыкновенной. Был картирован 131 маркер, в том числе 14 ортологичных, вы- явленных у всех изученных представителей семейства Ро- зоцветные. Позднее появилась работа по созданию генети- ческой карты малины западной (Bushakra et al., 2015a). Для картирования использовали популяцию из 115 сеянцев F1 комбинации ORUS 3021-2 × ORUS 4153-1. Один из роди- телей был устойчив к тле – Amphophora agathonica, что позволило локализовать на карте ген устойчивости Ag4. Первоначально карта содержала 566 полиморфных мар- керов. Позднее она была насыщена 468 SNP-маркерами, распределенными по семи псевдохромосомам, и сопостав- лена с физической картой (Jibran et al., 2018). При этом показана высокая степень коллинеарности генетической и физических карт малины, а также геномов малины и земляники. Устойчивость малины западной к тле изуча- лась и далее; были картированы три гена, ответственных за данный признак (Bushakra et al., 2018).

Еще одна карта была построена на основе анализа по- пуляции потомков скрещивания межвидового гибрида [R. idaeus (сорт ‘Tulameen’) × R. parvifolius] × сорт малины ‘Qualicum’ с использованием 161 AFLP- и 17 SSR-маркеров (Molina-Bravo et al., 2014). В этом исследовании определены участки, ответственные за адаптацию растений к низким температурам и колебаниям зимних тем- ператур (LG1, LG4, LG5 и LG6), а также за размер плода (LG5), его форму (LG6), цвет (LG1, LG5), за окраску вен- чика (LG5 и LG6) и плотность шипов (LG4 и LG6).

Для ежевик первая карта была сконструирована в 2013 г. (Castro et al., 2013) на основе анализа сегрегации в популяции потомков скрещивания сортов ‘Arapaho’ и ‘APF-12’. Участок, ответственный за отсутствие шипов, локализован в группе сцепления LG4, а QTL, контроли- рующий тип плодоношения, – в LG7.

**MAS (marker-assisted selection).** Несмотря на то что у разных видов малин и ежевик картированы многие гены и QTL селекционно-ценных признаков и определены тесно сцепленные с ними маркеры, статей по маркер-вспомога- тельной селекции известно крайне мало.

Большое значение для селекции имеет разработка мар- керов гена Bu, контролирующего устойчивость к вирусу RBDV – крайне опасному патогену, наносящему большой экономический ущерб. Для решения этой задачи J.A. Ward et al. (2012) применили метод сегрегационного bulk-анализа при помощи RAPD-маркеров. Специфичные для устойчивых генотипов фрагменты (BC002-900, BC296- 425, BC615-600) были секвенированы. На основе полу- ченных последовательностей созданы CAPS-маркеры, из которых наилучшую связь с устойчивостью показал маркер BC615_553_Alu I, отсутствующий, однако, у других устойчивых сортов. Далее последовательность этого маркера была выравнена по секвенированному геному земляники, при этом один из генов-кандидатов оказался гомологичен последовательности N-гена табака, контролирующего устойчивость к вирусу табачной мозаи- ки. В результате был предложен внутригенный маркер raspN_gene_1202, который в 96.7 % случаев связан с устойчивостью к RBDV. Эффективность данного маркера подтверждена и при анализе расширенной группы селек- ционных сортов: диагностический фрагмент выявлен у устойчивых сортов (‘Nootka’, ‘Haida’, ‘Willamette’, WSU 78117-1, ‘Newburgh’, ‘Cowichan’, ‘Chilcotin’, ‘Malling Promise’, ‘Latham’) и отсутствовал у поражаемых RBDV генотипов (Ward et al., 2012).

С целью разработки маркеров генов устойчивости к фитофторозной гнили корней J. Graham с коллегами (Graham et al., 2011) отобрали ВАС-клоны с фрагмента- ми хромосом 3 и 6, на которых ранее были картированы QTL устойчивости к Phytophthora rubi. Специфичных маркеров для QTL устойчивости к фитофторозной гни- ли корней получено не было, однако авторы отметили ассоциацию с этим признаком SSR-маркера Rub118b110 (Graham et al., 2011). В исследованиях C.A. Weber et al. (2008), направленных на решение этой же задачи, обна- ружены два основных QTL, связанных с устойчивостью к фитофторозной гнили корней. К данным участкам авторы создали SCAR- и CAPS-маркеры, которые при апробации на 18 сортах продемонстрировали уровень ассоциации с устойчивостью 76 %.

## Заключение

Современный инструментарий молекулярно-генетиче- ских методов применяется как в фундаментальных, так и в прикладных исследованиях представителей многочислен- ного рода Rubus, хотя число вовлеченных в исследования видов пока еще невелико (см. Приложение)1. Наиболее важным результатом в практическом плане является создание насыщенных молекулярно-генетических карт разных видов малин и ежевик, на которых локализованы многочисленные гены и QTL, детерминирующие хозяй- ственно ценные признаки. В то же время на сегодня для молекулярного скрининга доступно лишь небольшое чис- ло маркеров, разработанных для единичных генов. Благо- даря развитию современных технологий секвенирования, которые начинают применяться и для представителей рода Rubus, в ближайшее время можно ожидать прогресс и в направлении маркер-вспомогательной селекции.

## Conflict of interest

The authors declare no conflict of interest.
